# Nerve-sparing robotic-assisted radical prostatectomy: how I do it after 15.000 cases

**DOI:** 10.1590/S1677-5538.IBJU.2022.99.03

**Published:** 2021-11-10

**Authors:** Marcio Covas Moschovas, Vipul Patel

**Affiliations:** 1 AdventHealth Global Robotics Institute Celebration FL USA AdventHealth Global Robotics Institute, Celebration, FL, USA

## Abstract

**Introduction:**

Over the years, since Binder and Kramer described the first Robotic-assisted Radical Prostatectomy (RARP) in 2000, different Nerve-sparing (NS) techniques have been proposed by several authors ( 1 ). However, even with the robotic surgery advantages, functional outcomes following RARP, especially erection recovery, still challenge surgeons and patients ( 2 , 3 ). In this scenario, we have described different ways and grades of neurovascular bundle preservation (NVB) using the prostatic artery as a landmark until our most recent technique with lateral prostatic fascia preservation and modified apical dissection ( 4 - 6 ). In this video compilation, we have illustrated the anatomical and technical details of different grades of NVB preservation.

**Surgical technique:**

After the anterior and posterior bladder neck dissection, we lift the prostate by the seminal vesicles to access the posterior aspect of the prostate. Then, we incise the Denonvilliers layers and work between an avascular plane to release the posterior NVB from 5 to 1 and 7 to 11 o'clock positions on the right and left sides, respectively6. In sequence, we access the prostate anteriorly by incising the endopelvic fascia bilaterally (close to the prostate) until communicating the anterior and posterior planes. Finally, we control the prostatic pedicles with Hem-o-lok clips and then proceed for the apical dissection preserving the maximum amount of urethra length and periurethral tissues.

**Considerations:**

Potency recovery following radical prostatectomy remains a challenge due to its multifactorial etiology. However, basic concepts for nerve-sparing are crucial to achieving optimal outcomes, such as minimizing the amount of traction used on dissection, avoiding excessive cautery, and neural preservation based on anatomical landmarks (arteries and planes of dissection).

**Figure:** Available at:http://www.intbrazjurol.com.br/video-section/20229903_Patel_et_al
